# Profile of specific and associated autoantibodies in patients with idiopathic inflammatory myopathies in a Colombian population

**DOI:** 10.3389/fmed.2022.954937

**Published:** 2022-08-15

**Authors:** Andrés Hormaza-Jaramillo, Vanessa Bedoya-Joaqui, Germán Puerta-Sarmiento, Mario Bautista, Lady J. Rios-Serna, Tatiana Delgado-Mora, Ivana Nieto-Aristizábal, Ingrid Ruiz-Ordoñez

**Affiliations:** ^1^Unidad de Reumatología, Fundación Valle del Lili, Cali, Colombia; ^2^Unidad de Reumatología, Departamento de Medicina Interna, Facultad de Ciencias de la Salud, Universidad ICESI, Cali, Colombia; ^3^Centro de Investigación en Reumatología, Autoinmunidad y Medicina Traslacional, Universidad ICESI, Cali, Colombia; ^4^Centro de Investigaciones Clínicas, Fundación Valle del Lili, Cali, Colombia

**Keywords:** idiopathic inflammatory myopathies, myositis, autoantibodies, biomarkers, laboratory tests

## Abstract

**Objectives:**

Idiopathic inflammatory myopathies (IIMs) are chronic, autoimmune diseases with several forms of presentation. Diagnosis is mostly clinical in our region. Our aim was to evaluate the autoantibody profile of patients with IIMs.

**Methods:**

This study is a cross-sectional study with a prospective recollection of data, conducted between 2019–2021, in a single center in Cali, Colombia. Patients with a clinical diagnosis or suspicion of IIM were included. The presence of myositis-specific/associated antibodies was evaluated by immunoblotting in serum samples. Phenotypic characterization was performed.

**Results:**

A total of 36 patients were included. The mean age was 50.6 (16.7) years, and 20 (55.6%) were female. Eighteen (50%) patients were seropositive, of which 11 (30.5%) presented one positive antibody, with anti-TIF1ɣbeing the most frequent (*n* = 4, 11.1%), followed by anti-Ro52 (*n* = 2, 5.6%). Seven patients (19.4%) showed >1 positive antibody. Dermatomyositis was the most frequent type of IIM in seropositive patients (*n* = 8, 44.4%), followed by anti-synthetase syndrome (*n* = 4, 22.2%). Weakness was symmetric and presented in the upper and lower extremities in 11 (61.1%) patients each. Both respiratory insufficiency and weight loss were seen in 7 (38.9%) patients, Gottron papules in six (33.3%) patients, and heliotrope rash, esophageal dysmotility, and myalgia in 5 (27.8%) patients. Pulmonary interstitial disease was seen in 4 (22.2%, with antibodies for anti-Ro52, anti-MDA5 + anti-Jo1 + anti-TIF1ɣ, anti-MDA5 + anti-SAE1 + anti-NXP2, and anti-cN1A + anti-Ro52) patients, and malignancy was seen in 2 (11.1%) patients (1 with anti-Mi2β and 1 with anti-TIF1ɣ + anti-Mi2α). In all, 7 (19.4%) patients required intensive care (2 seropositive, 1 with anti-PL7, 1 with anti-MDA5 + anti-Jo1 + anti-TIF1ɣ), and 1 (2.8%) (seronegative) patient died.

**Conclusion:**

This study is the first study in the Southwest of Colombia that evaluates myositis-specific/associated antibodies in IIM. Half of the patients were seropositive. Anti-TIF1ɣwas the most frequent MSA and anti-Ro52 was the most frequent MAA. Several patients presented antibody combinations. Further studies are needed to fully associate phenotypes with antibodies.

## Introduction

Idiopathic inflammatory myopathies (IIMs, also known as myositis) are a heterogeneous group of autoimmune disorders characterized by several clinical phenotypes, histological changes, and autoantibodies, resulting in muscle damage, weakness, and inflammation. Dermatomyositis (DM) and polymyositis (PM) were the first pathologies recognized, followed by inclusion body myositis (IBM) (1, 2). Currently, PM would be destined to disappear as a disease to be replaced by a muscular inflammatory process with well-defined histological characteristics that is associated with myositis with autoimmune diseases, myositis due to specific autoantibodies, early stages of IBM, necrotizing myopathy immune-mediated (IMNM), and antisynthetase syndrome (ASS) (3).

A major advance in the field of IIM was the discovery of autoantibodies found in myositis patients, such as myositis-specific autoantibodies (MSAs, present in approximately 60–80% of IIM patients) or myositis-associated autoantibodies (MAAs), which are useful to establish a distinctive pattern of disease or phenotype with diagnostic, prognostic, and therapeutic implications (3–5). MSA [anti-Mi2 (also known as chromodomain-helicase-DNA-binding proteins), anti-melanoma-associated gene 5 (anti-MDA5), anti-nuclear matrix protein 2 (anti-NXP2), anti-transcriptional intermediate Factor 1γ (anti-TIF1γ), anti-small ubiquitin-like modifier activating enzyme (anti-SAE), anti-signal recognition particle (anti-SRP), anti-3-hydroxy-3-methylglutaryl-CoA reductase (anti-HMGCR), and anti-aminoacyl-tRNA synthetase (anti-ARS) such as anti-Jo1 (anti-histidyl), anti-PL12 (anti-alanyl), anti-PL7 (anti-threonyl), anti-OJ (anti-isoleucyl), anti-EJ (anti-glycol), anti-KS (anti-asparaginyl), anti-YRS/Ha (anti-tyrosyl), and anti-Zo (anti-phenylallyl)] are associated with a unique clinical phenotype, and these autoantibodies are found almost exclusively in patients with IMNM myopathy or ASS (6). MSAs are particularly useful in identifying patients at risk for interstitial lung disease (ILD) and malignancy (7–12). Therefore, it makes them a useful tool to optimize follow-up for these patients (3). MAAs [anti-Ro/SSA 52 kD, anti-Ku, anti-U1RNP, anti-PM/scleroderma autoantigen (PM/Scl), and anti-cytosolic 5′-nucleotidase 1A (cN1A)] are associated with both IMNM myopathies and other rheumatic diseases (6). Sometimes an MAA can occur together with an MSA (3, 13–15).

In our region, there is little information on IIM, and the diagnosis is based mainly on clinical findings. Currently, the anti-Jo1 antibody is the most frequently available in our population. There is a gap in knowledge regarding anti-Jo1-negative IIMs and their respective phenotypes. This article focuses on a phenotypic characterization associated with the autoantibody profile identified in patients with IIMs.

## Materials and methods

### Study design and participants

We conducted a cross-sectional study with prospective recollection from September 2019 to December 2021 at Fundación Valle del Lili, a high complexity center in Cali, Colombia. Adult and juvenile IIM [American College of Rheumatology (ACR)/European League Against Rheumatism (EULAR) classification criteria] (16), from hospitalization or ambulatory services were included, and a serum sample was obtained from each patient to detect the presence of myositis-specific/associated antibodies. A phenotypic characterization of the autoantibody profiles was performed. The medical records at the time of serum sample collection were evaluated to identify demographic, clinical, laboratory, treatment, and prognostic characteristics. Written informed consent to participate and to provide biological samples was obtained from all participants. This study complied with the principles described in the Declaration of Helsinki and was approved by the Institutional Ethics Committee of Fundación Valle del Lili (protocol number 1285).

### Detection of myositis-specific/associated antibodies

The presence of myositis-specific/associated antibodies against cN1A, Ro52, OJ, EJ, PL12, PL7, SRP, Jo1, PM/Scl75, PM/Scl100, Ku, SAE1, NXP2, MDA5, TIF1γ, Mi2α, and Mi2β was evaluated by immunoblotting using an anti-myositis antigen EUROLINE-WB kit (Euroimmun, Lubeck, Germany) according to the manufacturer’s instructions. Results were obtained as negative, positive (+,++), or solid positive (+++). Patients in whom positivity for >1 antibody were included, and those with only 1+ were discarded.

### Statistical analyses

A descriptive analysis was performed using Stata version 14 (StataCorp., College Station, TX, United States). Qualitative variables are summarized as absolute frequencies and proportions, and quantitative variables are summarized as the mean (SD) or median (IQR) according to Shapiro–Wilk’s test for normality.

## Results

### General characteristics

A total of 36 patients with a clinical diagnosis or suspicion of IIM were included. The mean age at inclusion was 50.6 (±16.7) years, and the majority were women (*n* = 20, 55.6%). The mean age at disease onset was 47.2 (±19.6) years, and the median duration of the disease was 9 (5–26) months. Several autoimmune diseases were found in association with IIM. Sjögren’s syndrome and hypothyroidism were the most frequent, seen in three (8.3%) cases each. Laboratory parameters were characterized by elevated creatinine phosphokinase, liver enzymes, and lactate dehydrogenase. Electromyography and muscle biopsy were performed on 36.1 and 41.7% of the participants, respectively. The most frequent finding in muscle biopsies was inflammatory infiltrates (86%, 13/15). Only 25% (2/8) presented with muscular edema on nuclear magnetic resonance (Table 1).

**TABLE 1:** Demographic and general characteristics.

Characteristics	*n* = 36 (*n*,%)
Female	20 (55.6)
Age at admission	50.6 (16.7)*
Age of onset	47.2 (19.6)*
Disease duration (months)	9 (5–26)**
**History of autoimmunity**	
Sjögren’s syndrome	3 (8.3)
Hypothyroidism	3 (8.3)
Systemic lupus erythematosus	2 (5.6)
Rheumatoid arthritis	2 (5.6)
Scleroderma	1 (2.8)
Autoimmune hepatitis	1 (2.8)
Discoid lupus	1 (2.8)
Chronic cutaneous lupus	1 (2.8)
Myasthenia gravis	1 (2.8)
Psoriasis	1 (2.8)
**Laboratory parameters, *n* (%)**	
Leucocytes	7.50 (6.21–11.61)**
Neutrophils	5.32 (3.87–9.49)**
Lymphocytes	1.32 (0.88–1.92)**
Platelets	294 (230–356)**
GSR	11 (4–34)**
CRP	0.46 (0.23–4.08)**
Creatinine	0.64 (0.48–0.84)**
Blood urea nitrogen	14.8 (10.1–17.5)**
AST	54.6 (39.6–171)**
ALT	64.8 (23–139.9)**
LDH	334 (245–628.5)**
CPK	593.5 (125–3594)**
Aldolase	11.2 (5.1–51.6)**
Electrophoresis performed	16 (44.4)
Monoclonal peak	2
Muscle biopsy performed	15 (41.7)
Inflammatory infiltrate	13
Variation in fibers’ size	8
Perifascicular atrophy	6
Nuclear internalization	5
Muscle necrosis	3
HLA I	2
Rimmed vacuoles	2
Electromyography	13 (36.1)
Myotatic pattern	8
Muscle MR performed	8 (22.2)
Muscle edema	2

*Mean (±SD); *Median (IQR); n, number; GSR, globular sedimentation rate; CRP, C reactive protein; AST, aspartate aminotransaminase; ALT, alanine aminotransferase; LDH, lactate dehydrogenase; CPK, creatinine phosphokinase; HLA, human leukocyte antigens; MR, magnetic resonance.

### General findings on muscle autoantibody profile

**FIGURE 1:**
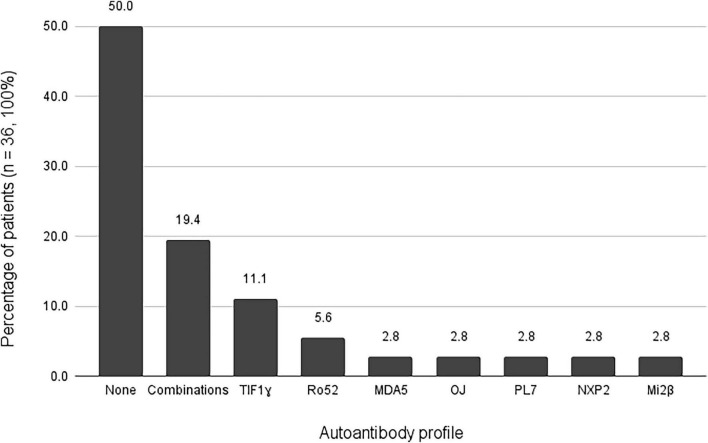
Findings in the muscle autoantibody panel.

Eighteen (50%) patients were seronegative. Of the seropositive patients (*n* = 18, 50%), eleven presented with only one positive antibody, with anti-TIF1ɣbeing the most frequent in 4 (11.1%) cases, followed by anti-Ro52 in 2 (5.6%) patients. Anti-MDA5, OJ, PL7, NXP2, and Mi2β were present alone in one (2.8%) patient each. Seven (19.4%) patients were found to have >1 positive antibody in different combinations. By dividing autoantibodies into MSA (anti-SRP, anti-SAE1, anti-NXP2, anti-MDA5, anti-TIF1γ, anti-Mi2α, anti-Mi2β, anti-OJ, anti-EJ, anti-PL12, anti-PL7, and anti-Jo1) and MAA (anti-cN1A, anti-Ro52, anti-PM75, anti-PM100, and anti-Ku), four patients had more than one MSA (anti-MDA5 + anti-Jo1 + anti-TIF1ɣ, anti-MDA5 + anti-SAE1 + anti-NXP2, anti-TIF1ɣ + anti-Mi2α, and anti-Mi2β + anti-Mi2α), two patients had a combination of MSA with MAA (anti-SAE1 + anti-PM/Scl75, and anti-NXP2 + anti-Ro52), and one patient had more than one MAA (anti-cN1A + anti-Ro52) (Figure 1).

### Clinical characteristics by autoantibody profile

The most frequent type of IIM was DM (*n* = 14, 36.8%). Of the seronegative patients (*n* = 18, 50%), four (22.2%) were classified as having DM, two (11.1%) were classified as having IBM, eight (44.4%) remained unclassified, and others were present to a lesser extent. Of the seropositive patients (*n* = 18, 50%), ten (55.5%) presented with DM, divided into four who had positive anti-TIF1ɣ, one with anti-Ro52, one with anti-NXP2, one with anti-Mi2β, and three with combinations of anti-MDA5 + anti-SAE1 + NXP2, anti-TIF1ɣ + anti-Mi2α and anti-Mi2β + anti-Mi2α. Four (22.2%) presented with anti-synthetase syndrome, of which one had anti-Ro52, one had anti-OJ, one had anti-PL7, and one had anti-MDA5 + anti-Jo1 + anti-TIF1ɣ. Two (11.1%) patients presented amyopathic dermatomyiositis, of whom one was positive for anti-MDA5, and the other was positive for anti-SAE1 + anti-PM/Scl75. Other types of IIM were diagnosed less frequently (Table 2).

**TABLE 2:** Autoantibody profile regarding the type of myopathy.

Type of myopathy	Seronegatives	Seropositives	TIF1ɣ	Ro52	MDA5	OJ	PL7	NXP2	Mi2β	MDA5 + Jo1 + TIF1ɣ	MDA 5+ SAE1 + NXP2	TIF1ɣ+ Mi2α	Mi2β + Mi2α	SAE1 + PM/Scl75	NXP2 + Ro52	cN1A + Ro52

	*n* = 18, 50%	*n* = 18, 50%	*n* = 4, 11.1%	*n* = 2, 5.6%	*n* = 1, 2.8%	*n* = 1, 2.8%	*n* = 1, 2.8%	*n* = 1, 2.8%	*n* = 1, 2.8%	*n* = 1, 2.8%	*n* = 1, 2.8%	*n* = 1, 2.8%	*n* = 1, 2.8%	*n* = 1, 2.8%	*n* = 1, 2.8%	*n* = 1, 2.8%
Dermatomyositis (DM)	4 (22.2)	10 (55.5)														
Juvenile DM	0 (0)	1 (5.6)														
Amyopathic DM	0 (0)	2 (11.1)														
Unclassified	8 (44.4)	1 (5.6)														
Polymyiositis	1 (5.6)	0 (0)														
Antisynthetase syndrome (ASS)	0 (0)	4 (22.2)														
Inclusion body myositis (IBM)	2 (11.1)	0 (0)														
Necrotizing myopathy immune-mediated (IMNM)	1 (5.6)	0 (0)														
Genetic	1 (5.6)	0 (0)														
Idiopathic	1 (5.6)	0 (0)														

This table shows the type of myopathy by seronegativity and seropositivity and discriminates by each profile found. The dark cells show what mypathy was present for each profile. For TIF1ɣand Ro-52, all patients presented with dermatomyositis (DM).

In terms of clinical presentation (Table 3), in the seronegative patients (*n* = 18, 50%), the main characteristic was lower extremity weakness in 16 (88.9%), followed by symmetric weakness in 15 (83.3%) cases. Myalgia was seen in seven patients (38.9%), and esophageal dysmotility was seen in six patients (33.3%). In the seropositive patients (*n* = 18, 50%), weakness was symmetric and was present in the upper and lower extremities in 11 (61.1%) patients. Respiratory insufficiency and weight loss were found equally in seven (38.9%) patients, Gottron’s papules were found in six (33.3%), and heliotrope rash, esophageal dysmotility, and myalgia were present in 5 participants (27.8%). artralgia, and ILD were found in four (22.2%) patients each.

**TABLE 3:** Autoantibody profile by clinical presentation.

Clinical presentation	Seronegatives *n* = 18, 50%	Seropositives *n* = 18, 50%	TIF1ɣ *n* = 4, 11.1%	Ro52 *n* = 2, 5.6%	MDA5 *n* = 1, 2.8%	OJ *n* = 1, 2.8%	PL7 *n* = 1, 2.8%	NXP2 *n* = 1, 2.8%	Mi2β *n* = 1, 2.8%	MDA5 + Jo1 + TIF1ɣ *n* = 1, 2.8%	MDA5 + SAE1 + NXP2 *n* = 1, 2.8%	TIF1ɣ+ Mi2α *n* = 1, 2.8%	Mi2β + Mi2α *n* = 1, 2.8%	SAE1 + PM/Scl75 *n* = 1, 2.8%	NXP2 + Ro52 *n* = 1, 2.8%	cN1A + Ro52 *n* = 1, 2.8%
**Weakness**																
Symmetric	15 (83.3)	11 (61.1)	3													
Upper extremities	13 (72.2)	11 (61.1)	4													
Lower extremities	16 (88.9)	11 (61.1)	3													
**Signs and symptoms**																
Respiratory insufficiency	2 (11.1)	7 (38.9)														
Heliotrope rash	2 (11.1)	5 (27.8)	2													
Gottron’s papules	3 (16.7)	6 (33.3)	3													
Gottron’s sign	0 (0)	4 (22.2)	1													
Shawl sign	3 (16.7)	3 (16.7)	1													
Pistolera sign	0 (0)	3 (16.7)	1													
Mechanic’s hands	2 (11.1)	1 (5.6)														
Digital ulcers	0 (0)	1 (5.6)														
Raynaud	1 (5.6)	2 (11.1)														
Esophageal dysmotility	6 (33.3)	5 (27.8)	2													
Myalgia	7 (38.9)	5 (27.8)	2													
Artralgia	3 (16.7)	4 (22.2)	2													
Weight loss	4 (22.2)	7 (38.9)	2													
Malignancy	1 (5.6)	1 (5.6)														
Interstitial lung disease	1 (5.6)	4 (22.2)														

This table shows the clinical characteristics regarding seronegativity, seropositivity, and discrimination by each profile found. The dark cells correspond to the characteristics that were present in each profile. For TIFɣ, it is specified how many of the four patients presented with each characteristic. For Ro-52, each characteristic was present in one of two patients.

#### Interstitial lung disease

Interstitial lung disease was seen in one (5.6%) patient with no antibodies, corresponding to non-specific interstitial pneumonia, and in four (22.2%) seropositive patients, as follows: two had non-specific interstitial pneumonia (one with positive anti-Ro52, one with anti-MDA5 + anti-Jo1 + anti-TIF1ɣ), one had usual interstitial pneumonia and presented with positive anti-cN1A + anti-Ro52, and one had an unclassified ILD and presented with positive anti-MDA5 + anti-SAE1 + anti-NXP2 (Table 3).

#### Malignancy

Malignancy was seen in two (5.6%) patients from the whole sample. Papillary thyroid carcinoma was seen in one (5.6%) seronegative patient, and lung small-cell carcinoma was seen in one (5.6%) patient with anti-TIF1ɣ + anti-Mi2α (Table 3).

#### Prognosis

Of the hospitalized patients, one (5.6%) patient with an undefined type of IIM, who was seronegative, died. Five (27.8%) seronegative and two (11.1%) seropositive patients (one with anti-PL7 and one with anti-MDA5 + anti-Jo1 + anti-TIF1ɣ) required admission to the intensive care unit (ICU). No patient required lung transplantation (Table 3).

### Treatment

Treatment was evaluated in 36 patients, and we found that almost all patients (*n* = 35, 86.1%) received steroids, most of them prednisolone (*n* = 24, 66.6%), and the median weekly dosage was 15 (5-50) mg. Azathioprine was the second most used drug, found in 15 (41.7%) patients, followed by rituximab in 5 (13.9%) patients who received one cycle each. Cyclophosphamide, cyclosporine, methotrexate, and intravenous immunoglobulin therapy were used in four participants each (11.1%). Combination therapy was used in three patients (azathioprine + cyclosporine, azathioprin + methotrexate, and mycophenolate mophetil + cyclosporine).

## Discussion

Our study identified the presence of specific autoantibodies associated with myositis in 50% of the participants. This finding is similar to that reported in the literature, where the presence of these autoantibodies has been reported in 53 to 80% of patients with IIM (3–5, 17). It is important to highlight the usefulness of autoantibody associations to establish a phenotypic pattern with diagnostic, prognostic, and therapeutic implications in IIM. This study is the first study in the Southwest of Colombia to describe phenotypic characteristics of autoantibody profiles and is the second to report the frequency of myositis-associated/specific autoantibodies (18). When faced with a patient with IIM, two different perspectives could be taken. The first is to determine, according to the clinical diagnosis, the frequency of autoantibodies present for each disease and their consequent relative risk of other comorbidities. The second is to establish, in the presence of one or more autoantibodies, the probability of the development of one or more different diseases, determine their phenotypic characteristics, predict the risk of comorbidities with high morbidity and mortality, and provide a timely and personalized therapeutic approach. Could it be that diagnosing clinically is enough to know the true systemic compromise that a patient with IIM has or could have in the future?

In our study, it was found that the majority of affected people were women (55.6%, 20/36), similar to what is described in most autoimmune conditions. DM, PM, and ASS were more frequent in women than in men (55%, 11/20 vs. 37.5%, 6/16), consistent with what has been reported in the literature (19). More than 50% of the men presented with autoantibody-specific and/or autoantibody-associated myositis. Combinations of autoantibodies were more frequent in men than in women (55.5%, 5/9 vs. 22.2%, 2/9). The autoantibodies with the highest prevalence in women were anti-TIF1ɣand anti-Ro52. IIM may be associated with other autoimmune diseases, such as scleroderma, systemic lupus erythematosus, rheumatoid arthritis, Sjögren’s syndrome, and mixed connective tissue disease. The frequency with which autoimmune muscle disease occurs in the context of other connective tissue diseases has not been well defined (6). A total of 44.4% of all our participants had associated rheumatic disease. Sjögren’s syndrome together with autoimmune hypothyroidism were the most frequent autoimmune connective tissue diseases (8.3%, 3/16 each). In the case of Sjögren’s syndrome, the percentage of patients who manifest symptoms of myositis varies widely between 0.6 and 10% (20–23).

Idiopathic inflammatory myopathies can affect any age group, from early childhood to late adult life. The mean age of onset in our study was 47.2 years (±19.6), similar to that reported in Latin America (18, 24) and lower than that reported in France and Greece (25, 26). Both age over 45 years and male sex have been the demographic parameters most frequently associated with an increased risk of malignancy in patients with IIM (27). In our study, the risk of malignancy was found in patients over 65 years of age, and the combination of anti-TIF1ɣ + anti-Mi2α autoantibodies was reported in a patient with small cell carcinoma of the lung. Ovarian, breast, and lung cancer have been reported as major malignancies in patients with IIM (28). Therefore, MSA plays a fundamental role in characterizing the clinical phenotype and prognosis (29).

Of the 17 autoantibodies measured in our population, only five were not detected, and several combinations of them were present (38.9%, 7/18). Previous studies have shown that up to half of patients have combinations of autoantibodies (17). Some studies have reported that myositis-specific antibodies are mutually exclusive (30) and rarely coexist (31). In other studies, it has been described that although autoantibodies are related to a certain subtype of IIM, they can also be present in different sub-classifications and co-exist with others. (32). Suzuki et al. proposed discriminating antibodies into four groups: those associated with DM, ASS, IMNM, and other autoantibodies (33). In our cohort, no combination of autoantibodies was repeated, evidencing the wide spectrum of autoantibodies present in our population and their associated heterogeneity of clinical presentation.

Most of the autoantibodies studied were associated with the development of DM in any of its sub-classifications (classic DM, juvenile DM, or amyopathic DM). In Latin America, DM has been reported as the most prevalent IIM subtype (18, 24, 34). The most frequent MSA in our study was anti-TIF1ɣ(11.1%, 4/18), whereas in Argentina, it was anti-Jo1 (16.1%), followed by MDA5 (10.8%) and Mi2 (10.2%) (24). In 6 Latin American countries, including Colombia, it has been reported that the most frequent MSAs were anti-Mi2 (38.5%) and anti-Jo1 (11.9%) (18).

The anti-TIF1ɣautoantibody has been detected as a predictor of cancer and has been associated with severe skin involvement (35, 36). We found that patients with positivity only for this antibody developed classic DM with significant skin involvement, the same as reported in other studies (32, 37). Only one of our participants developed malignancy. Age seems to play a fundamental role in the risk of malignancy, and adults under 40 years of age seem not to be at higher risk (11). However, it could be that our anti-TIF1ɣ-positive patients had not yet developed malignancy at the time of the study. Anti-TIF1ɣhas been reported more frequently in Caucasian than in Asian populations (38). This autoantibody was the most frequent in our population and its frequency was similar to the Caucasian population (38).

In our population, the presence of anti-Mi2 alone or in combination with other MSAs was reported in 16.6% (3/18) of patients and was associated with development of DM. Similar observation has been reported in the literature, mainly in young patients (39). A very varied prevalence of this antibody has been described, between 2 and 45% (40). In the United States and Canada, the prevalence of anti-Mi2 was lower than that reported in Central and South America (18, 40). Furthermore, differences in prevalence have been found within the same country. For example, the prevalence varies from 5 to 27% in Italy, and from 2 to 19% in Japan (38). These differences in prevalence between countries and within the same country could be related to both geographic location and autoantibody detection technique.

In the patients Mi2 positives in our study, Shawl’s sign, Gottron’s sign, Gottron’s papules, alopecia, symmetrical weakness in four limbs, myalgia, esophageal dysmotility, and fever were evident. In patients with combined antibodies including anti-TIF1ɣ, respiratory muscle weakness, respiratory failure, weight loss, digital ulcers, and one case of malignancy were also found. It is difficult to determine whether the additional systemic involvement is due to positivity for anti-Mi2 or anti-TIF1ɣor a combination of these antibodies. Although it is consistent with some studies where the presence of anti-Mi2 has been related to the characteristic clinical development of DM, they have been generally associated with a good prognosis, low risk of ILD, and good survival (32, 39–41). None of our patients with this autoantibody developed ILD, required ICU admission or lung transplantation, or died.

The anti-MDA5 autoantibody is part of the group of patients associated with DM according to the Suzuki classification (33). In our study, a case with amyopathic DM was presented, and ILD developed in combination with other MSAs. A relationship between amyopathic DM and rapidly progressive ILD has been reported (40, 42). Its prevalence in different studies has ranged from 3 to 58% of patients with myopathies and has been shown in 100% of patients with amyopathic DM. However, these studies have been conducted primarily in Asian and American countries where differences between populations have been observed (40, 43). Anti-MDA5 was more frequent in Asian than in Caucasian populations (38). Our findings showed a lower frequency of presentation than in China (32) and a frequency similar to the Caucasian population (38).

Patients with positivity for anti-NXP2 were diagnosed with classic DM and juvenile DM. In other studies, it has been described as associated with juvenile DM with greater frequency (35, 40, 42, 44). The prevalence of anti-NXP-2 in DM varies from 1.6 to 30% (45–47). In a cohort of Argentine patients with pediatric myositis, anti-NXP-2 antibodies were reported to be the most prevalent (25% of cases) (48). In our investigation, a low frequency of this antibody was found, which could be due to a low frequency of patients with juvenile DM. The only patient with juvenile DM was positive for anti-NXP2 in combination with anti-Ro52. It was found to be the only positive antibody in a patient with skin involvement. NXP2-positive cases have been reported to more frequently show typical manifestations of facial DM as a heliotrope rash (*p* < 0.0001; OR 3.4, 95% CI 1.88–6.2) (49). Like many MSAs, its prevalence is highly variable, as is its association with the development of cancer (35, 42). This association could be due to ethnicity, environmental factors, and age. The association with cancer has been found in Japanese population but was not found in Italian population (38). In our cohort, no patient developed a neoplasm similar to that reported in other reports (32).

Anti-SAE1 antibodies have been associated with a high frequency of skin lesions, such as heliotrope rash, Gottron’s rash, and dysphagia (40). As in our study, previous research has described a low frequency of anti-SAE1 antibodies (50–53). In our anti-SAE1 findings, positivity was not reported as a single autoantibody, which makes it difficult to determine its association with a characteristic phenotype. When associated with an MAA, respiratory failure, heliotrope rash, Gottron’s papules, Gottron’s sign, Shawl’s sign, and holster sign were present. In the presence of other MSAs, respiratory failure, weight loss, and ILD occurred. In a Japanese cohort, anti-SAE1 positivity was associated with the development of ILD (50). In some cases, its association with cancer has been reported (54). However, in our population, no patient with this antibody developed cancer.

Anti-ARS autoantibodies such as anti-Jo1, anti-PL7, and anti-PL12 were reported in our study to be associated with ASS. Anti-Jo1 has been described as the most common anti-ASS, and other anti-ASSs have been reported to have a prevalence of 0.5 to 6% (17, 24, 40). In our series, a higher frequency was observed (16.7%, 3/18), as in Argentina (16.1%) (24). A high frequency of anti-PL12 has been found in the United States and of anti-PL7 in Japanese population (39). The ASS is generally characterized by some combination of ILD, arthritis, myositis, and cutaneous findings such as mechanic’s hands. Several recent studies suggested heterogeneity in clinical characteristics among different patients with anti-ARS antibodies (30, 40). In our case, the subjects with positive anti-OJ and anti-PL7 developed symmetric muscle weakness in the upper and lower limbs, PL7 was associated with weight loss, anti-OJ was associated with respiratory failure, and none had ILD, whereas positivity of the antibody anti-Jo1 was present in a patient with arthralgia, respiratory failure, and development of ILD. Patients with anti-PL7 and anti-Jo1 antibodies required ICU management, which could be associated with greater disease severity in these patients. In the literature, anti-PL7 and anti-PL12 have been associated with more prevalent and severe ILD (41). Anti-Jo1 has been linked to more muscle involvement and arthritis than the other anti-ASS antibodies (39, 55). Survival was not altered in our patients, contrary to what has been reported (30).

Anti-SRP antibody was not found in our series, which may be explained by the low frequency of appearance in patients with IIM (35). In addition, it has been related to the appearance of anti-SRP syndrome (severe necrotizing myopathy) (56), a syndrome that was not reported in our population. In our study, IMNM necrotizing myopathy was detected in only one patient (2.8%), which is lower than what was reported in a study in China (37).

Of the MAAs, the most common was anti-Ro52, similar to what has been reported in previous studies (57). A higher frequency of severe myositis, joint involvement, ILD, and cancer with poor prognosis have been reported, especially if it is associated with anti-ASS antibodies (36, 41, 58, 59). Although it has also been associated with anti-MDA5 (60) and anti-SRP (61). In our study, one patient with Ro52 + and NXP2 + developed symmetric muscle weakness in the lower limbs and another patient with Ro52 + and cN1 + developed respiratory failure, ILD, Raynaud’s phenomenon, myalgia, and arthralgia. ILD was present in two of our Ro52 + patients, consistent with that described by other authors (13, 15). However, none required ICU or lung transplantation. No deaths or cancer were reported in these patients.

Anti-cN1A autoantibodies have been related to IBM, juvenile DM, Sjögren’s syndrome, and systemic lupus erythematosus (30, 62). In our investigation, it was only found in one patient with rheumatoid arthritis and positive anti-Ro52. The anti-Ku autoantibody has been found in 13, 2, and 1% of patients diagnosed with PM/SSc overlap syndrome, PM, and DM, respectively (63). Perhaps due to the low presence of this MSA in patients with DM and PM, it was not found in our population. Something similar was found with anti-PM/ScL-75 and anti-PM/ScL-100 autoantibodies. In the literature it has been reported that anti-PM/Scl autoantibodies were found in 17% of patients with PM/SSc overlap syndrome, 6% of PM patients, and 9% of DM patients (63). In our patients, there was a low frequency of PM and systemic sclerosis, which could influence a low frequency of anti-PM/ScL-75 and the absence of anti-PM/ScL-100. The widely varying preva–lence of these antibodies have been established in different ethnicities (64).

There were differences between data reported in Latin America, other continents, and our research. For example, in Mexico anti-Mi2 has been reported as the most prevalent antibody (65), similar to what has been informed in PANLAR Myositis Study Group (18). In addition, this antibody has been demonstrated in one in patients from Brazil diagnosed with PM or DM. In our population, anti-TIF1ɣwas the most frequent MSA and anti-Ro52 was the most frequent MAA. Results similar to those found in other studies were obtained. For example, in Brazil anti-Ro52 was the most frequent MAA in patients diagnosed with PM or DM (66). A common genetic risk factor HLA-DRB1*0701 has been shown in anti-Mi2 + European and American patients with DM (44). In our population, the frequency of this histocompatibility complex is unknown.

The variability in the frequency of MII autoantibodies worldwide has not only been related to ethnicity, environmental and genetic factors, and age but also geographic location, and autoantibody detection technique. Geographic latitude has been reported to be an important factor in the prevalence of some autoantibodies. It has been described that anti-Mi2 + increased closer to the Equator meanwhile anti-NXP2 + and anti-ARS + antibodies had an opposite behavior, increasing in the geographical locations farther to the Equator (65). However, studies have been reported where a significant increase in anti-Mi2 frequency toward the Equator was not attributed (44). There was no difference in the frequency of these antibodies in our study.

Concerning the technique for detecting autoantibodies in IIM, the need for international standardization and optimized methods for wider distribution has been reported in the literature to improve reproducibility and patient stratification. Different laboratory techniques have been used for its processing, such as indirect immunofluorescence, ELISA (enzyme-linked immunoassay), and Western blot. The most objective way to characterize the reactivity of the antibodies is the search for the antigen-antibody reaction, with different techniques of immunoprecipitation of radiolabeled proteins or RNA molecules, but due to the slow processing, the large number of antigens required, and the low sensitivity, this method has not been a routine procedure in most clinical laboratories. (44). In recent years, autoantibody panels have been proposed that allow several of them to be measured together, so linear immunoassay could become a technique to be taken into account within standardization given its good operating characteristics when compared to immunoprecipitation (“gold standard”). Yoo IS, et al. (38), reported that comparing conventional classification systems, seroclinical classification could designate patients in the subgroup more appropriately. Furthermore, according to Stuhlmüller, et al. (44), in future studies it is important to conduct research to improve the staging of patients with IIM according to their antibody profile with respect to the response to different treatment options. We agree with these authors, there are currently several criteria to classify patients without an adequate consensus to group them. As observed in our findings, immunological profile and clinical features of the patients are very varied. It is imperative to standardize a method to detect a complete panel of autoantibodies in MII in order to generate better comparisons between different phenotypes. Moreover, each antibody predisposes to the development of multiple symptoms and one symptom can be developed by multiple antibodies, so the response to treatment of each patient could be different in patients presenting with similar symptoms. Having an adequate grouping of patients with a clinical serological concept, studies of response to treatment could be carried out to guide future conduct in clinicians. However, this is something that is beyond the scope of our study, so further study is required in subsequent studies.

On the other hand, it has been reported that anti-SRP autoantibodies seem to correlate with clinical activity, anti-Jo1 were associated with disease activity, and in some patients with remission of the disease. It has been shown that anti-MDA5 autoantibodies decrease or disappear when the disease was in remission (38, 44). In our research, this parameter was not evaluated, but we agree that studying these characteristics could help improve patient care since it would allow better control of the clinical conditions of patients. In addition, among the other reasons previously mentioned, this could contribute to the variability in the frequency of autoantibodies reported, since not in all cases was it specified whether the patient had disease activity at the time of study entry. Therefore, the evaluation of autoantibodies at different moments in the natural history of the disease could help clinicians determine the aggressiveness of treatment to avoid undesirable outcomes.

Among the limitations of our study is the use of a single method for detecting autoantibodies, which is why some of them could not have been detected. Therefore, an underreporting of their frequency in the different pathologies could have been generated. On the other hand, in the test used for the detection of autoantibodies, there was no detection of the anti-HMG-Coenzyme A reductase antibody, and it was not possible to establish its frequency. Anti-U1-RNP was also not detected. Additionally, given that the clinical information was obtained from medical records, not all variables were available for all patients.

## Conclusion

In our study, we describe the profile of antibodies present in patients with idiopathic inflammatory myopathy and the clinical characteristics developed with each immunological profile. We identified autoantibodies specific to and associated with myositis in 50% of the patients. Anti-TIF1ɣwas the most frequent MSA and anti-Ro52 was the most frequent MAA. Multiple patients presented with combinations of antibodies. Further studies are needed to fully associate the phenotypes with autoantibodies

## Data availability statement

The datasets generated and/or analyzed during this study are not publicly available in order to protect patient information. Further inquiries can be directed to the corresponding author.

## Ethics statement

The studies involving human participants were reviewed and approved by Comité de Ética en Investigación Biomédica Fundación Valle del Lili. The patients/participants provided their written informed consent to participate in this study.

## Author contributions

AH-J: substantial contributions to the conception and design of the work, and interpretation of data. VB-J: substantial contributions to the analysis and interpretation of data for the work. GP-S: substantial contributions to the design of the work and the acquisition data. MB: substantial contributions to the design of the work and the acquisition data. LR-S substantial contributions to the acquisition and interpretation of data for the work. TD-M and IR-O: substantial contributions to the acquisition of data for the work. IN-A: substantial contributions to the acquisition and analysis of data for the work. All authors contributed to the article and approved the submitted version.
